# Advanced lung cancer patient benefits from minimally invasive costal resection and reconstruction: an effective palliative approach for costal metastasis

**DOI:** 10.1186/s13019-023-02422-y

**Published:** 2023-11-10

**Authors:** Marco N. Andreas, Dirk Böhmer, Johann Pratschke, Jens C. Rückert, Aron Elsner

**Affiliations:** 1 Department of Surgery, Charité – Universitätsmedizin Berlin, Corporate Member of Freie Universität Berlin and Humboldt-Universität Zu Berlin, Augustenburger Platz 1, 13353 Berlin, Germany; 2 Department of Radiation Oncology, Charité - Universitätsmedizin Berlin, Corporate Member of Freie Universität Berlin and Humboldt-Universität Zu Berlin, Hindenburgdamm 30, 12203 Berlin, Germany

**Keywords:** Minimally invasive, Video-assisted thoracoscopy (VATS), Rib resection, Chest wall reconstruction

## Abstract

We hereby describe the resection and reconstruction of a rib infiltrated by a lung cancer metastasis. Despite prior radiation therapy aimed at mitigating pain from rib infiltration in a stage IV non-small cell lung cancer patient, results were unsatisfactory. Employing a minimally invasive palliative strategy, we executed a successful operation to address this issue. This technique presents a viable alternative for patients experiencing recurrent pain post radiation therapy.

**Supplementary Information:**

The online version contains supplementary material available at 10.1186/s13019-023-02422-y.

## Background

Non-small cell lung cancer (NSCLC) stands as a significant contributor to global mortality [1]. Among these patients, 30–40% exhibit bone metastases, with ribs being the prevailing site of implantation [2]. Radiation therapy serves as the primary treatment to alleviate pain resulting from tumor infiltration. However, when pain resurfaces post-radiation, available options become limited. A recent systematic review demonstrated the potential of metastasectomy in alleviating pain for patients with bone metastasis [3].

The conventional method of performing rib resection or reconstruction typically involves a large opening, leading to substantial soft tissue damage. This approach not only results in unsightly scarring but also contributes to heightened postoperative pain and an extended hospitalization period.

Video-assisted thoracoscopy (VATS), a firmly established technique in thoracic surgery, offers a potential solution. Applying the principles of VATS to chest wall surgery, as indicated by the accounts of specific cases, holds promise for a more refined approach [4–6]. Ilhan et al*.* reported a successful case of minimally invasive rib resection for fibrous dysplasia. This accomplishment was achieved through uniportal thoracoscopy [4]. Contemporary research highlights that rib resection as a sole intervention for thoracic wall defects can result in complications such as lung herniation and chronic pain syndrome. These findings underscore the necessity of incorporating defect reconstruction following rib resection [7].

This case report illustrates that both rib resection and reconstruction, serving as a palliative treatment to alleviate pain in patients with bone metastasis, can be executed with minimal invasiveness, resulting in excellent cosmetic outcomes and effective postoperative pain reduction.

### Case presentation

**Fig. 1:**
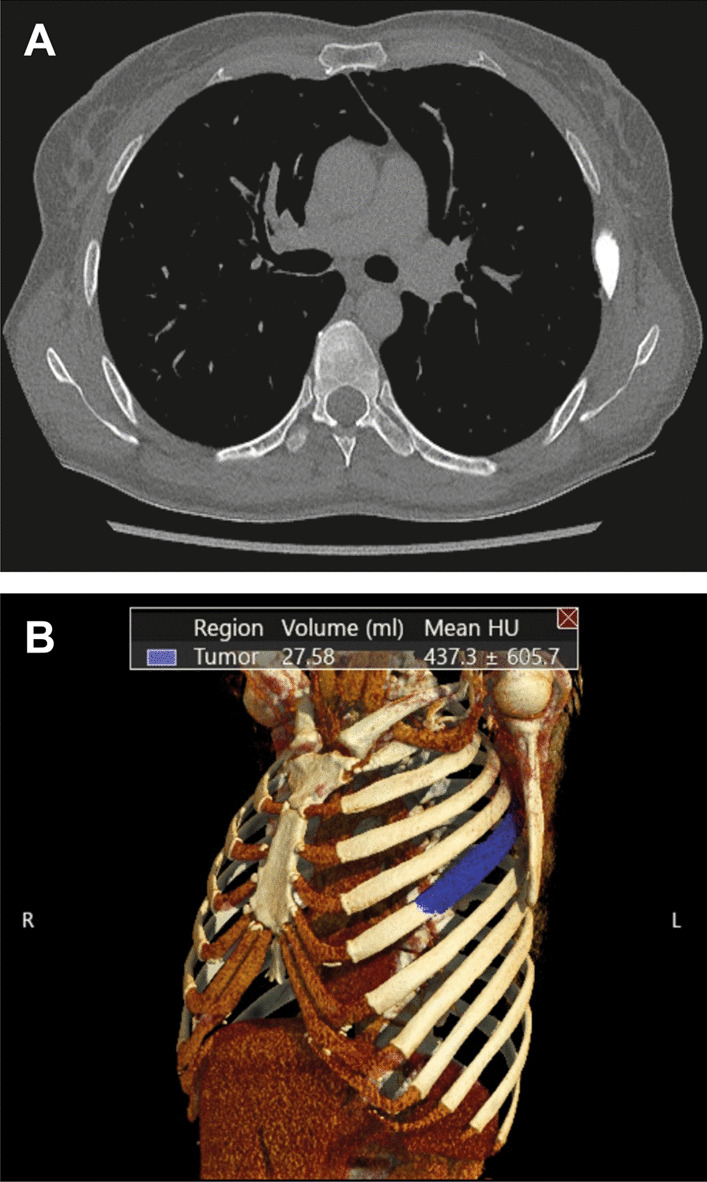
**A** The axial CT scan vividly displays the expansion of the 5th rib metastasis in NSCLC. **B** In the CT reconstruction of the NSCLC metastasis, the tumor is delineated in blue, and its volume is quantified (27.58 ml)

A 47-year-old female patient diagnosed with advanced adenocarcinoma of non-small cell lung cancer (NSCLC) presented to our outpatient clinic. Four years had elapsed since the initial diagnosis, during which time the tumor had metastasized to the brain (n = 4), the cervical spine, and the left 5th rib (Fig. 1).

She had previously undergone kyphoplasty to address the spine metastasis and had received multiple rounds of whole brain irradiation. At the point of surgical evaluation, her overall physical condition was favorable (ECOG 9-10), aside from intermittent bouts of dizziness. The primary factor compromising her quality of life was chronic pain, notably stemming from the tumor infiltration of the 5th rib. Despite previous attempts at pain relief through radiation and immune therapy, the pain persisted and even worsened rapidly (Visual Analogue Scale, VAS, preoperatively = 8). Preoperative assessments revealed no abnormalities in blood count or biochemistry. As a palliative therapeutic strategy, we proposed a minimally invasive total rib resection and reconstruction, considering the patient's condition.

### Surgical technique

The patient was positioned in the right lateral decubitus with both arms raised. General anesthesia was induced, and a double-lumen tube facilitated single lung ventilation.

A 3 cm incision was made in the anterior axillary line, directly over the 5th rib. Another incision was created paravertebrally, also spanning the 5th rib. Intercostal muscles were carefully displaced using a retractor through both access points. The metastatic rib was identified through the anterior incision by inserting a 30° thoracoscope (ENDOEYE, Olympus, Hamburg, Germany). Once the affected rib was located, the separation of intercostal muscles was extended along the rib using the retractor, as previously mentioned. The rib was then excised, with the dorsal section cut approximately 1 cm ventral to the Processus transversus, and the ventral part trimmed shortly after the costochondral junction using rib scissors. The excised rib was sent for pathological examination. Remarkably, there were no indications of pleural adhesions or fibrosis, despite the patient's prior radiation therapy.

**Fig. 2:**
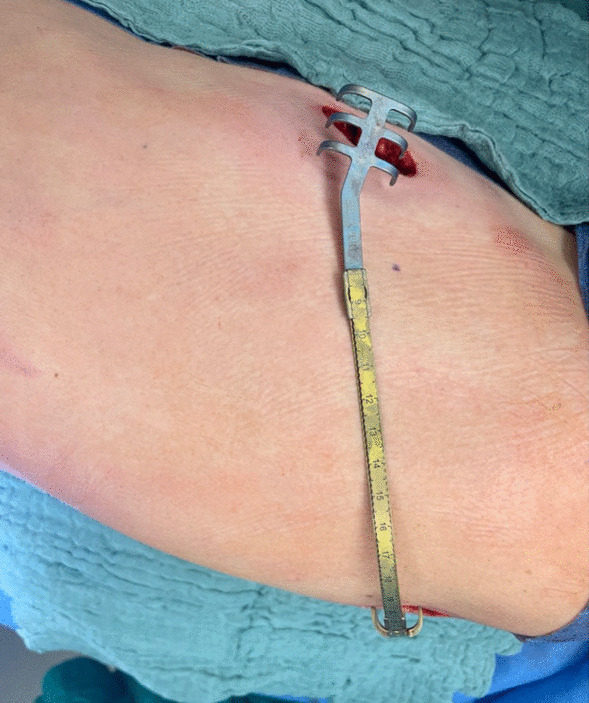
Two rib clips (one equipped with a rotatable connector) and a connection bridge (MedXpert, München, Germany) are externally connected and shaped to conform to the thoracic wall. The upper part of the image features the ventral incision, while the lower image section partially shows the dorsal incision positioned under the second rib clip

For thoracic wall reconstruction, two rib clips (one with a rotatable connector) and a connecting bridge (MedXpert, München, Germany) were employed (Fig. 2).

**Fig. 3:**
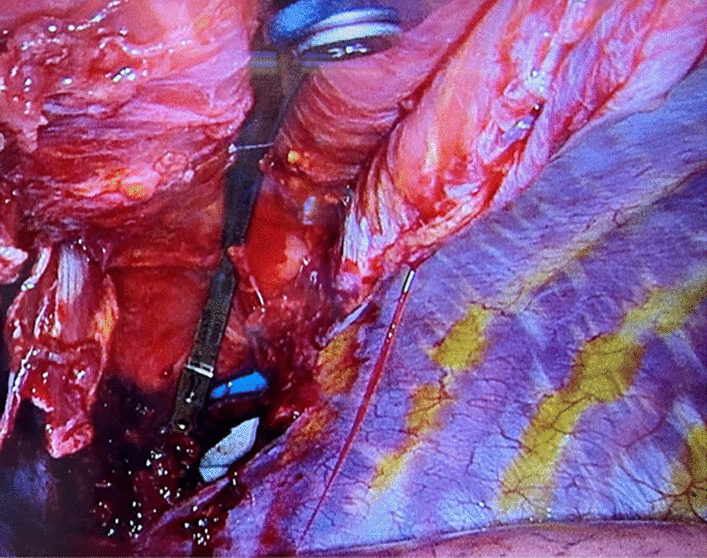
Taken from an intrathoracic perspective, this image illustrates the rib replacement already positioned atop the two rib stumps, centrally visible

Preoperatively, the implant components were tailored to the patient's anatomical features using CT images as a reference. The assembled construct was externally connected and bent to mimic the resected rib, then introduced into the thoracic cavity through the ventral incision. Under thoracoscopic guidance, the construct was securely positioned and anchored to the remaining rib stumps (Fig. 3).

The fixation was achieved without screws, a distinct feature of the manufacturer's devices to minimize discomfort caused by screws. Instead, the backside of the rib clips incorporated multiple spikes that could be bent around the rib stumps and subsequently linked to the connection bridge. A 20 Charriere drainage tube was inserted, and both incisions were meticulously closed layer by layer using absorbable sutures. A suction of 8 cmH_2_O was applied.

**Fig. 4:**
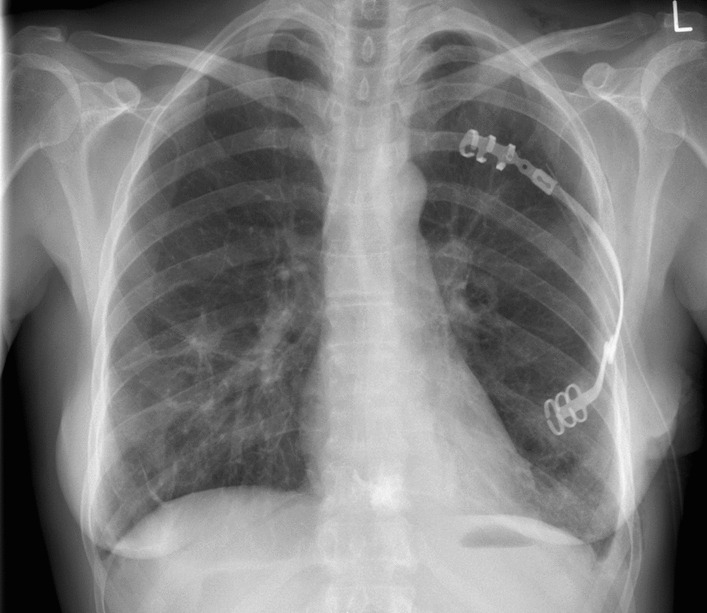
The postoperative X-ray provides a clear view of the implant's placement

The total duration of the procedure was 2 h and 34 min, with extubation taking place in the operating room. On the first postoperative day, the patient reported a pain level of 8 on the Visual Analog Scale. A follow-up X-ray revealed successful placement of the implant (Fig. 4).

**Fig. 5:**
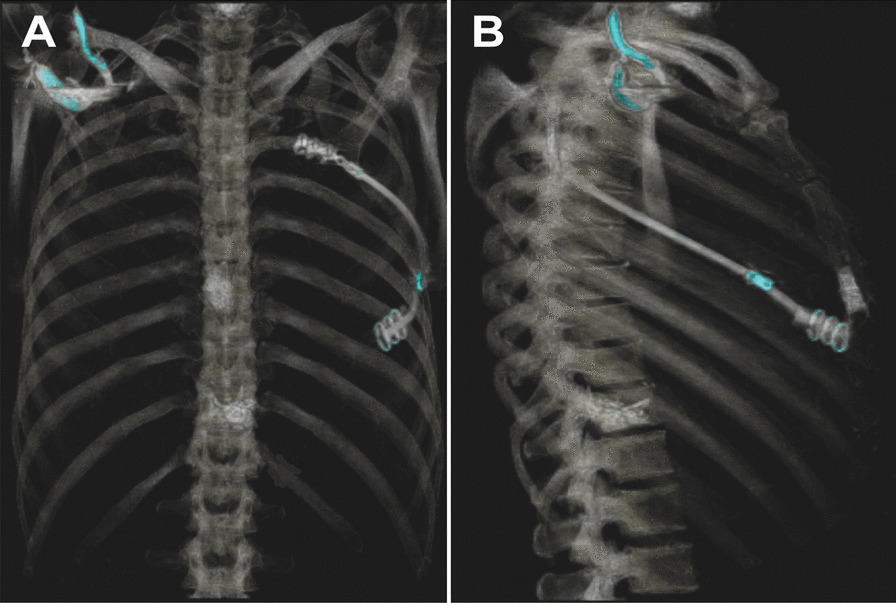
After a span of 12 months, we conducted a CT scan revealing an uncompromised position of the implant. The figure depicts the CT reconstruction, illustrating the implant's placement from a posterior-anterior view (**A**) and from the left side (**B**)

The drainage was removed on the same day. Managing postoperative pain for this patient necessitated a comprehensive approach, including patient-controlled anesthesia (PCA). The PCA was withdrawn on the third day. The patient was discharged seven days after the procedure, reporting a VAS of 4. After a span of 12 months, a CT scan was conducted, confirming the implant's secure position without any indications of displacement (Fig. 5).

## Comment

Metastases of NSCLC that extend to the ribs can induce intense pain and significantly diminish one's quality of life [2]. Therapeutic avenues for these patients become limited if the tumor remains unresponsive to radiation. As a palliative strategy to alleviate pain, resection of the affected rib emerges as a viable option. Simultaneous reconstruction serves to prevent lung herniation and mitigate the risk of chronic pain [7].

Opting for a VATS procedure as opposed to an open approach not only diminishes postoperative pain but also leads to a shortened hospital stay and improved aesthetic outcome, attributable to minimized soft tissue damage [8]. This aligns with contemporary advancements in thoracic surgery, particularly in the realm of thoracic wall reconstruction, where a trend toward minimally invasive approaches is evident. Although our operation's duration may seem relatively lengthy, it still falls within the lower range of time durations when compared to existing literature [9]. Additionally, the expedited recovery following the procedure cannot be overstated, particularly for patients grappling with tumors. This philosophy is congruent with fast-track surgery concepts aimed at reducing overall complications by minimizing immobilization periods [10].

We also emphasize the significance of evading post-thoracotomy syndrome and thoracic wall numbness in metastatic cancer patients, as these issues can further erode the quality of life, which is already compromised, for these individuals. While one might argue that scarring holds less significance for those contending with advanced cancer, and only survival matters, this particular case underscores a distinct viewpoint. The patient, despite her grave illness, explicitly conveyed a strong preference for minimal scarring. This preference carried weight for her, serving as a source of positivity and a semblance of normalcy. As a comparatively young woman conscious of her body, she aspired to be able to present herself, perhaps at a beach, without an overtly conspicuous scar.

In conclusion, our findings indicate that rib reconstruction via VATS can be executed safely, yielding favorable aesthetic outcomes and facilitating swift reintegration into social life. This approach stands as a palliative therapeutic strategy for rib metastasis.

### Supplementary Information

**Additional file 1: Table S1** Pre- and postoperative pain medication

## Data Availability

Data will be made available on request.
