# Supplementary material for: Advanced lung cancer patient benefits from minimally invasive costal resection and reconstruction: an effective palliative approach for costal metastasis
Source: J Cardiothorac Surg. 2023 Nov 10;18:310. doi: 10.1186/s13019-023-02422-y (PMC10636914; doi:10.1186/s13019-023-02422-y)
Supplement: Supplementary file 1 — Additional file 1: Table S1 Pre- and postoperative pain medication [file 13019_2023_2422_MOESM1_ESM.docx]

**Table 1 – Pre- and postoperative pain medication**

| Pre-  operation |  |  | Post- operation |  |  |
| --- | --- | --- | --- | --- | --- |
| Morphine retard | 10 mg | 2-1-1 | Morphine retard | 30 mg | 1-1-1 |
| Morphine | 10 mg | bolus 8 - 10 times / day | Morphine | 10 mg | no need for bolus |
| Ibuprofen | 400 mg | 1-1-1 | Ibuprofen | 400 mg | 1-1-1 |
| Metamizole | 500 mg | 1-1-1 | Metamizole | 500 mg | 1-1-1 |
